# Hidden Mystery behind Unilateral Cerebellar Infarction in Hanging: A Case Report

**DOI:** 10.7759/cureus.31115

**Published:** 2022-11-05

**Authors:** Ruchita Kabra, Shilpa A Gaidhane, Mansi Patel, Pratik J Bhansali, Sourya Acharya, Sunil Kumar

**Affiliations:** 1 Internal Medicine, Datta Meghe Institute of Medical Sciences (Deemed to be University), Wardha, IND; 2 Department of Medicine, Jawaharlal Nehru Medical College, Datta Meghe Institute of Medical Sciences (Deemed to be University), Wardha, IND; 3 Radiodiagnosis, Datta Meghe Institute of Medical Sciences (Deemed to be University), Wardha, IND; 4 Department of Medicine, Jawaharlal Nehru Medical College, Wardha, IND

**Keywords:** infarction, hanging, compression, hypoxia, suicide

## Abstract

One of the most popular ways to commit suicide is by hanging. Injury after hanging typically results from pressure on the veins and arteries of the neck; airway compression is less likely, and cervical spine injuries are quite uncommon. Due to the severity of the hypoxic and ischemic brain injury, the external compression results in venous congestion of the brain, hypoxic circulation, diminished arterial supply to the brain, and possible death. Following a near-hanging, cerebral infarction or hypoxic encephalopathy without ischemia may be the mechanism causing brain damage.

## Introduction

One of the most popular suicide methods and a method used in death punishment is hanging [1]. The majority of case reports describe occlusion and dissection of the internal carotid artery in various degrees resulting in cerebral infarction in injuries for individuals who survive the hanging [2-6]. The most common cause of autopsied deaths is cerebral hypoxia rather than spinal cord damage, which is why treating hypoxia in individuals who have survived a hanging episode should be the first priority. The thalamus, cerebellum, and other regions of the basal nuclei have all been characterised as having bilateral haemorrhagic or ischemic lesions in hanging [5-9]. As they haven't yet been reported, unilateral lesions appear to be an extremely rare occurrence. This is a case of unilateral involvement of the cerebellum after hanging.

## Case presentation

A 38-year-old male who committed suicide by hanging was brought by his wife to the emergency department in an intubated state from a primary care hospital. The patient attempted to hang himself under the influence of alcohol intoxication.

**Figure 1:**
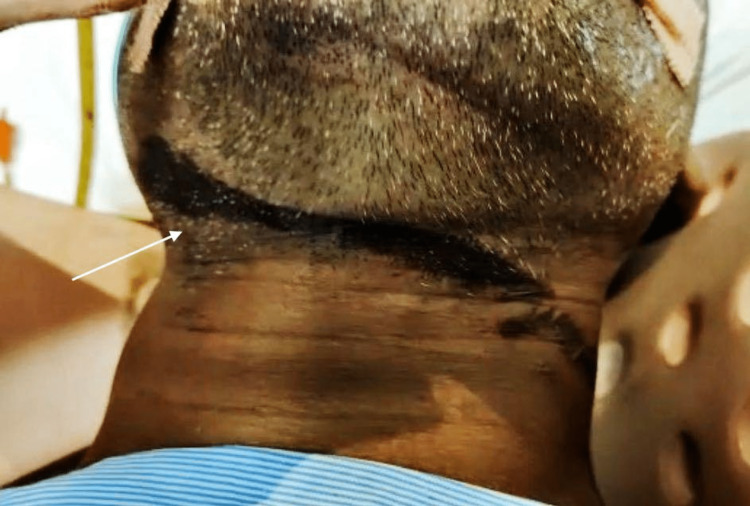
Ligature mark of hanging as shown with arrow

On initial evaluation, the patient was drowsy and in an intubated state with a respiratory rate of 12/min, and FiO2- 80% with a Glasgow Coma Score of 5/15 (E2VTM3). One circumferential superficial abraded ligature mark was visible upon local examination around the cricoid cartilage beginning at the right angle of the jaw and extending up to 10cm X 2.5cm (Figure 1).

Systemic examination revealed normal heart sounds in the cardiovascular system, bilateral equal air entry on respiratory examination, per abdomen soft and non-tender with no organomegaly; central nervous examination revealed a drowsy and intubated state.

The patient had a history of psychiatric illnesses like major depression and suicidal thoughts. Also, there was a history of attempted suicide one year ago by cuts on arm. Blood parameters were within normal limits and ABG was suggestive of metabolic acidosis.

X-ray of the cervical spine showed no fracture. Chest X-ray showed no abnormality and electrocardiogram suggestive of sinus rhythm; CT Brain was normal.

The patient was treated aggressively in the medicine intensive care unit with injectable antibiotics, methylprednisolone, and other supportive management. A cervical collar was attached. Later, he improved with a Glasgow coma scale of 9/15 (E4V1M4). The patient's vitals and saturation were monitored and he was extubated.

**Figure 2:**
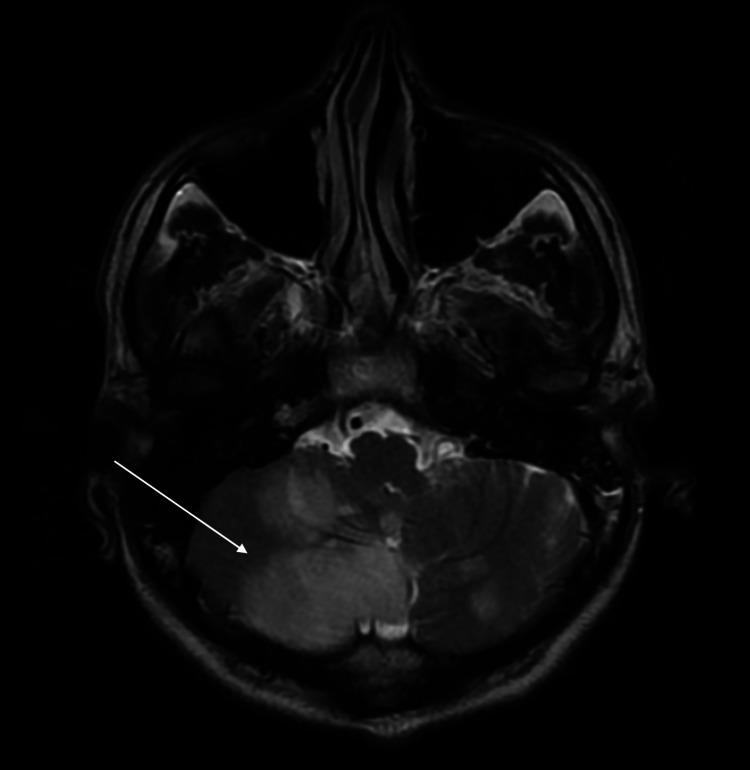
T2/FLAIR MRI shows hyperintensity (arrow) in the right cerebellar hemisphere and few foci in the left cerebellar hemisphere

**Figure 3:**
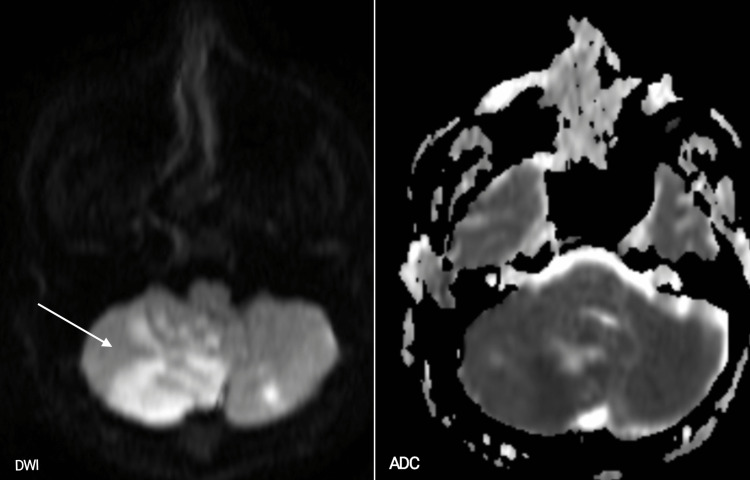
DWI MRI Image shows restriction (arrow) corresponding to low signals on ADC.

**Figure 4:**
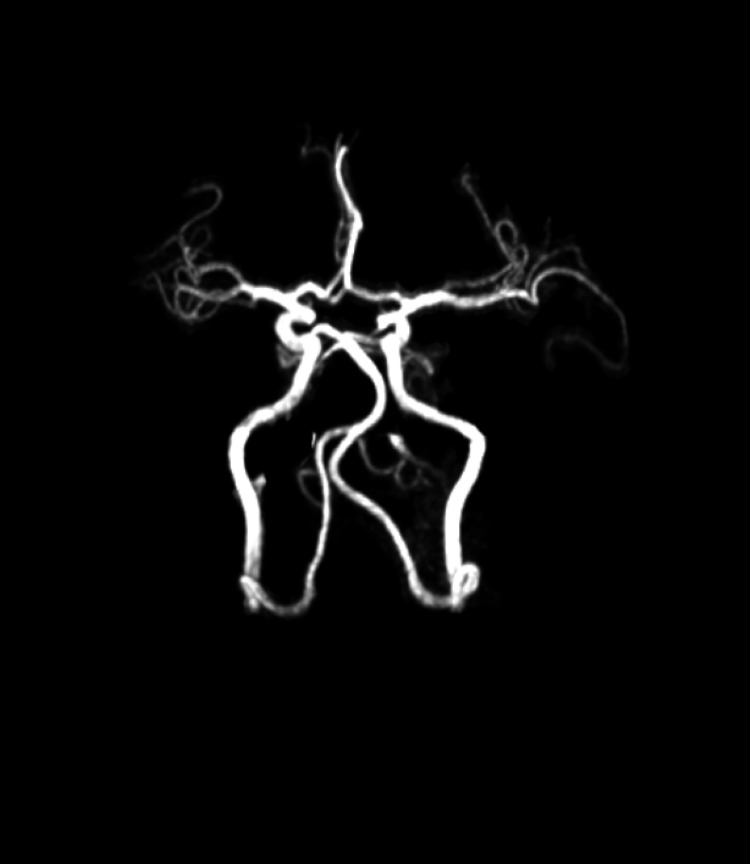
MR Angiography showing normal filling and no defect.

After stabilisation, MRI Brain with angiography was done which suggested acute infarcts in unilateral cerebellar hemispheres and vermis in the form of multiple areas of altered signal intensities predominantly involving the right cerebellar hemisphere, appearing hyperintense on T2W1/FLAIR MRI (Figure 2). DWI MRI showed restriction corresponding to low signals on ADC (Figure 3). MR Angiography was normal as shown in figure 4. The patient was given mannitol, dual antiplatelet, and other supportive management. The patient denied prior brain imaging. On the 10th day, the patient was discharged. On follow-up after 15 days, the patient was doing well and was continued with oral antiplatelets.

## Discussion

In developed nations during the 20th century, hanging has been a rare type of execution despite being one of the most popular methods of successful suicide [10]. In a legal hanging, the second and third cervical vertebrae are broken (Hangman's fracture), which is followed by an atlantoaxial dislocation that transections the pontomedullary and results in instantaneous death.

As hanging is the most common method of suicide and accounts for 72% of all suicides in India [11], various radiological findings such as diffuse swelling, symmetrical low-density areas in the medial thalami and lentiform nuclei, bilateral low-density areas in the globi pallidi, left and right parietal haematomas, and a little amount of subarachnoid haemorrhage (according to CT findings) are reported [5,6]. The above case report demonstrates that as opposed to the typically seen unilateral involvement, bilateral lesions can also be seen following a hanging incident with an ischemic-arterial event being the most likely source of these lesions. The pathologic repercussions of a hanging from a suicidal attempt and the legal hanging of capital punishment differ significantly. Cerebral anoxia and ischemia in a suicidal hanging develop more slowly, and both phenomena are greatly influenced by the materials, setting, and precise manner of the suicide attempt [2,5]. The factors that lead to death following suicidal hanging are varied. Mechanical compression and obstruction of the neck's airway and vasculature, upward displacement of the tongue and epiglottis, external pressure on the carotid sinuses and the resulting hyperactivity, and (in rare cases) direct injury to the spinal cord and brain stem, including vertebral artery damage, are all causes of cerebral hypoxia and ischemia [3]. 

Several papers have described particular CT alterations in hanging, and others have described MRI lesions. The lentiform nuclei, medial thalami, bilateral low-density areas in the globi pallidi, left and right parietal haematomas, diffuse edema, and a small amount of subarachnoid haemorrhage are among the CT findings [2,7-9].

According to published research, the length of hanging is correlated with the outcome, and various small studies have demonstrated that hanging lengths of under five minutes indicate a favourable outcome [12,13]. To our understanding, lesions are bilateral and symmetrical even when they hang for less than five minutes. As both arterial and venous MR angiography showed no filling defect and a normal cerebral venous system, it is unclear where the origins of the patient's lesions were in this case. The second scenario appears much less likely given his age.

The vertebro-basilar system may have experienced severe artery compression that caused a brief hypoperfusion as the mechanical dynamics of this patient's injury. Due to the lesion's unilateral placement, lack of hemorrhagic symptoms, and unilateral location, a venous infarction would have been unlikely. The patient's brain imaging was done on the sixth day after the injury during the subacute period. Therefore, it is likely that there were no areas of water restriction on the DWI. The distinct features of the lesions discovered through MR and CT studies may have also been caused in part by an anatomical variance that resulted in basal unequal perfusion inside the thalamic nuclei that was invisible to MR angiography.

## Conclusions

The multiple-reported bilateral position on the basal nuclei may not always correspond to cerebral lesions from hanging, which is vital for the diagnostician to keep in mind. The physician can determine the aetiology of the damage, their general strategy to treating the patient, and their prognosis with the help of knowledge of alternative imaging presentations of hanging patients.
